# Liver-Specific Extracellular Matrix Enables High-Fidelity Patient-Derived Hepatocellular Carcinoma Xenograft Models

**DOI:** 10.34133/bmr.0242

**Published:** 2025-08-21

**Authors:** Su Kyeom Kim, Jungho Bae, Mi Jeong Lee, Dai Hoon Han, Seung-Woo Cho

**Affiliations:** ^1^ Department of Biotechnology, Yonsei University, Seoul 03722, Republic of Korea.; ^2^ Department of Surgery, Yonsei University College of Medicine, Seoul 03722, Republic of Korea.; ^3^ Cellartgen, Seoul 03722, Republic of Korea.; ^4^ Center for Nanomedicine, Institute for Basic Science (IBS), Seoul 03722, Republic of Korea.

## Abstract

Patient-derived tumor xenograft (PDX) models serve as powerful tools in oncology research owing to their ability to capture patient-specific tumor heterogeneity and clinical behavior. However, the conventional matrices derived from murine tumors, commonly used to generate PDX models, suffer from key limitations such as lack of tissue specificity, high production costs, and inconsistent batch quality. In response, our study investigates the use of decellularized liver extracellular matrix (Liver ECM) as a biomimetic alternative that more accurately recapitulates the native hepatic microenvironment. We demonstrate that Liver ECM, enriched with liver-specific biochemical cues, enables robust engraftment, growth, and metastasis of patient-derived hepatocellular carcinoma cells in both subcutaneous and orthotopic PDX models. Notably, orthotopic models established with Liver ECM exhibited enhanced metastatic behavior, particularly to the intestine, compared to those formed using conventional matrices. Transcriptomic analysis further revealed activation of key pathways associated with cancer progression, including angiogenesis, apoptosis, migration, and inflammation. Additionally, we extend the application of Liver ECM to patient-derived organoid xenografts, which showed improved tumorigenicity and retained pathophysiological features of the original tumor tissue. Together, these findings underscore the potential of liver-specific ECM as a superior platform for generating physiologically relevant PDX models and enhancing the translational relevance of preclinical cancer studies.

## Introduction

The heterogeneity of human liver cancer poses substantial challenges to the effectiveness of drug treatments. To improve the precision of drug development, models that can recapitulate tumor phenotypes and capture accurate therapeutic responses are essential. Patient-derived xenograft (PDX) models, which are established by transplantation of human tumors into immunodeficient mice, have gained increasing attention in oncology research, drug discovery, and precision medicine [1,2]. These models preserve the genomic characteristics and clinical heterogeneity of cancer patients across various stages, molecular profiles, and therapeutic contexts, making them ideal for preclinical drug testing, validating new drug combinations, and investigating mechanisms of drug resistance [2,3]. Based on tumor transplantation site, PDX models are classified as either a subcutaneous or an orthotopic model. The subcutaneous model allows for straightforward monitoring of tumor size, thereby facilitating the rapid evaluation of drug efficacy. However, a subcutaneous environment differs substantially from that of the original tumor tissue, raising concerns about the model's ability to accurately replicate the complexities of human cancer. The orthotopic model supports tumor growth within the microenvironment of the target organ, facilitating tumor progression and metastasis [4]. However, technical challenges caused by the complexity of the transplantation procedure reduce the success rate and reproducibility of xenografts. Human patient-derived organoids (PDOs) have emerged as a promising patient-specific tumor model. The patient-derived organoid xenograft (PDOX) model offers several advantages over conventional xenograft systems. PDOs retain the intratumoral heterogeneity of primary tumors, enabling more accurate modeling of tumor biology and drug response. When transplanted in vivo, PDOX models more closely preserve the genetic, histological, and phenotypic characteristics of the parental tumor than conventional cell line-derived xenografts [5,6]. Compared to traditional PDX models, PDOs provide greater viability and are amenable to in vitro manipulation prior to engraftment, facilitating improved reproducibility, higher engraftment efficiency, and the generation of multiple experimental replicates from a limited amount of patient tissue [5,7]. Orthotopic transplantation further enables tissue-specific tumor progression, including metastatic behavior and microenvironmental interactions.

Liver cancer presents substantial challenges for drug development, largely due to its complex etiology and the absence of representative preclinical models [8]. Notably, the success rate of generating liver cancer organoids is around 30%, which is significantly lower compared to organoids from other tumor types [5,9,10]. This limited success is attributed to the fact that liver cancer organoids can only be derived from a narrow subset of liver cancer subtypes, with no clear correlation between successful organoid derivation and patients’ clinical characteristics [10]. Thus, reliable liver cancer models are urgently needed to better understand drug sensitivity and resistance across diverse liver cancer subtypes. Current PDX and PDO models rely on mouse sarcoma-derived matrices (e.g., Matrigel) enriched in laminin and proteoglycan components, which differ substantially in composition from liver tissues [11,12]. Accordingly, such matrices with non-liver-specific origin often fail to provide an adequate microenvironment for human liver cancer [11,13]. They may not provide tumorigenic microenvironments optimal for the patient-derived liver cancer cells and organoids, resulting in inaccurate patient cancer phenotype replication and low reproducibility in drug testing [11]. These situations underscore the need for alternative matrix that can better recapitulate a liver-specific microenvironment for advanced PDX models with pathophysiology of liver cancer.

Given the limitations of the current matrix, here we utilized a liver tissue-derived extracellular matrix (Liver ECM) to improve the existing liver cancer PDX models. Liver ECM derived from decellularized porcine liver tissue retains liver-enriched ECM components (e.g., COL6A1, COL6A2, COL6A3, ASPN, BGN, FBN1, and FGA) and various liver-specific proteins while cellular components are completely removed [11,14]. Therefore, Liver ECM is expected to provide a liver-specific microenvironment that can promote growth and differentiation of hepatocellular carcinoma (HCC) cells and liver cancer organoids more effectively than Matrigel and synthetic matrices [14–16]. Our findings demonstrate that Liver ECM (Regenix Liver) achieved tumorigenesis efficacy comparable to Matrigel in subcutaneous PDX models. Interestingly, Liver ECM outperformed Matrigel in orthotopic PDX models. Application of Liver ECM to orthotopic transplantation resulted in significantly higher tumor volumes, greater levels of differentiation, and increased metastasis. These results suggest that Liver ECM integrates with host liver tissue, potentiating tumorigenesis by enhancing cell–cell and cell–ECM interactions in the PDX models with patient-derived HCC cells and liver cancer organoids. Therefore, incorporating our Liver ECM scaffolds enhances the physiological relevance of PDX and PDOX models by providing biochemical and mechanical cues essential for recapitulating the hepatic tumor microenvironment (TME). Our study highlights the potential of tissue-specific ECM for efficient establishment of PDX models, facilitating reliable screening of anti-cancer drug candidates and precision medicine for patient-specific drug identification.

## Materials and Methods

### Cell culture

Human liver organoids, patient-derived HCC cells, and liver cancer organoids were isolated from patient liver tissues or patient tumors, which was approved by the Institutional Review Board (IRB) of Yonsei University Health System (Permit Number: 4-2016-0728). The healthy liver ductal cells and patient-derived HCC cells were isolated using previously reported protocols [5,17]. Briefly, patient liver tissues and tumors were finely chopped into small pieces and incubated using digestion enzyme comprising 0.125 mg/ml of Collagenase (#C9407; Sigma-Aldrich, St. Louis, MO, USA) and 0.125 mg/ml of Dispase II (#D4693; Sigma-Aldrich) in Dulbecco’s Modified Eagle Medium (DMEM) high glucose (#11995-073; Thermo Fisher Scientific, Waltham, MA, USA) supplemented with 1% (v/v) penicillin/streptomycin (P/S; #GIB-15140-122; Thermo Fisher Scientific) and 1% (v/v) fetal bovine serum (FBS; #26140079; Thermo Fisher Scientific) at 37 °C for 45 min. After enzymatic digestion, the supernatant from liver tissues and tumors was collected through 70- and 100-μm strainers, respectively. The residual tissue fragments were pipetted vigorously with basal medium (Advanced DMEM/F12 [#12634-010; Thermo Fisher Scientific] supplemented with 1% [v/v] P/S, 1% [v/v] HEPES [#15630-080; Thermo Fisher Scientific], and 1% (v/v) Glutamax [#35050061; Thermo Fisher Scientific]) to obtain high yields of liver ductal and cancer cells. The collected ductal and cancer cells were then centrifuged at 250 *g*, 4 °C for 5 min and resuspended in ACK lysing buffer (#A1049201; Thermo Fisher Scientific) to eliminate red blood cells. Subsequently, centrifuged liver ductal and HCC cancer cells (1.2 × 10^5^ cells) were encapsulated in 30 μl of growth factor reduced (GFR) Matrigel (#354230; Corning, NY, USA) or Liver ECM (Regenix; #RLI401, #RLI601; Cellartgen, Seoul, Korea) and then seeded in 48-well plates. For healthy liver organoids, liver organoid isolation medium was added for the first 3 days and replaced with liver organoid expansion medium every 2 days. Passaging was performed every 7 to 10 days. For liver cancer organoids, the isolation medium was refreshed every 2 or 3 days with passaging conducted after 21 days. The liver organoid expansion medium consisted of basal medium with 10% (v/v) R-spondin1-conditioned medium, B27 (#17504001; Thermo Fisher Scientific), N2 supplement (#17502-048; Thermo Fisher Scientific), 10 mM nicotinamide (#N0636; Sigma-Aldrich), 1 mM *N*-acetylcysteine (#A9165, Sigma-Aldrich), 50 ng/ml human EGF (#AF100-15; Peprotech, Cranbury, NJ, USA), 100 ng/ml human FGF10 (#100-26; Peprotech), 10 nM human gastrin I (#G9145; Sigma-Aldrich), 25 ng/ml human HGF (#100-39; Peprotech), 10 μM Forskolin (#F9929; LC laboratory, Gyeonggi-do, Korea), and 5 μM A83-01 (#2939; Tocris, Bristol, UK). The liver organoid isolation medium was the liver organoid expansion medium supplemented with 30% (v/v) Wnt3a-conditioned medium, 25 ng/ml human noggin (#120-10C; Peprotech), and 10 μM Rock inhibitor (#1293823; Biogems, Westlake Village, CA, USA).

### Quantitative real-time polymerase chain reaction

Liver and xenograft liver cancer samples were obtained from mouse subcutaneous and orthotopic xenograft models. These samples were immersed in lysis buffer and homogenized with a TissueLyser (Qiagen, Chatsworth, CA, USA). Total RNA was extracted using the Qiagen RNeasy Mini Kit (#74106; Qiagen), following the manufacturer’s protocol. The RNA was then reverse transcribed into complementary DNA (cDNA) with PrimeScript 1st strand cDNA Synthesis Kit (#6110A; TaKaRa Bio Inc., Shiga, Japan). Quantitative real-time polymerase chain reaction (qPCR) was performed using TaqMan Fast Universal PCR Master Mix (2×) (#4366073; Thermo Fisher Scientific) on a StepOne Plus Real-Time PCR System (#43-766-00; Applied Biosystems, Waltham, MA, USA). Target gene expression levels were measured by the comparative C_T_ (ΔΔC_T_) method and normalized to that of glyceraldehyde 3-phosphate dehydrogenase (GAPDH). The TaqMan primers used in this analysis included Alpha-fetoprotein (*AFP*) (Hs01040598_m1), Albumin (*ALB*) (Hs00910225_m1), Keratin 19 (*KRT19*) (Hs00761767_s1), Tumor necrosis factor alpha (*TNF-α*) (Hs00174128_m1), *Ki67* (Hs04260396_g1), Actin alpha 2 (*ACTA2*) (Hs00426835_g1), Cadherin-1 (*CDH1*) (Hs01023895_m1), Platelet-derived growth factor receptor beta (*PDGFRB*) (Hs01019589_m1), and *GAPDH* (Hs02786624_g1).

### Mouse xenograft models

All mouse experiments were approved by the Institutional Animal Care and Use Committee (IACUC) of Yonsei University under permit number (IACUC-A-202403-1831-01). The animals were maintained under controlled environmental conditions with a temperature of 21 ± 2 °C, a humidity of 50% ± 10%, ventilation of 10 to 15 air changes per hour, and noise levels maintained below 60 dB. The human HCC cell line (HepG2; #HB-8065; ATCC, Manassas, Virginia, USA), patient-derived HCC cells, and liver cancer organoids were used for subcutaneous and orthotopic transplantation in 7-week-old male Balb/c nude mice (Orient Biotech, Seongnam, Korea). In the subcutaneous xenograft model, each mouse received an injection of 1 × 10^6^ cells or 300 organoids mixed in 100 μl of Matrigel or Liver ECM (Regenix) into the right flank using a 31G insulin syringe (#328822; BD Ultra-Fine, Franklin Lakes, NJ, USA). For the orthotopic xenograft model, mice were anesthetized with ketamine (100 mg/kg, Yuhan, Seoul, Korea) and xylazine (40 mg/kg, Bayer Korea, Ansan, Korea), and 1:1 mixture of patient-derived HCC cells and hydrogel (Matrigel or Liver ECM) (total 20 μl containing 1 × 10^6^ cells) was injected into the median lobe of the liver using a 31G insulin syringe. Mice were euthanized by CO_2_ inhalation and liver cancer xenograft tissues were harvested from the mice at each point. To confirm intestinal metastasis in the liver cancer xenograft model, the orthotopically induced PDX models using Matrigel and Liver ECM were sacrificed after 12 weeks. Metastatic tumors were harvested, weighed, and carefully separated from normal intestinal tissue. The tumors were then fixed with 10% formalin and embedded in paraffin for subsequent histological and immunohistochemical analyses.

### Tumor volume measurement

Tumor volume was estimated noninvasively over time by measuring the shortest (width) and longest (length) diameters of the tumor bulge at the injection site using calipers. The formula used for tumor volume calculation was as follows: Volume = 0.5 × length × width^2^ (mm^3^).

### Histological and immunohistochemistry analysis

The tumor samples were fixed with 10% (v/v) formalin and embedded in paraffin for hematoxylin (#HCP-0100-00A; CellPath, Newtown, UK) and eosin (#230251, Sigma-Aldrich) (H&E) staining and immunohistochemical staining. For immunohistochemical staining, the tumor tissue sections were treated with antigen retrieval solution (citrate buffer, pH 6.0; #C9999; Sigma-Aldrich) and stained with the following primary antibodies: rabbit anti-ALB (#A3293, 1:200; Sigma-Aldrich), mouse anti-AFP (#SC8399, 1:100; Santa Cruz, Dallas, TX, USA), rabbit anti-Ki67 (#ab15580, 1:200; Abcam, Cambridge, UK), mouse anti-α-smooth muscle actin (α-SMA) (#SC53142, 1:100; Santa Cruz), rat anti-CD11b (#ab8878, 1:200; Abcam), mouse anti-CHGA (#SC393941, 1:100; Santa Cruz), rabbit anti-KRT19 (#4558S, 1:200; Cell Signaling, Danvers, MA, USA), rabbit anti-PDGFRB (#3169T, 1:200; Cell Signaling), anti-human GAPDH (#MA5-50219, 1:200; Thermo Fisher Scientific), anti-human nuclear antigen antibody (#ab191181, 1:200; Abcam), mouse anti-ECAD (#14472, 1:200; Cell Signaling), mouse anti-KRT18 (#MA5-12104, 1:200; Thermo Fisher Scientific), and mouse anti-MUC2 (#SC15334, 1:100; Santa Cruz). Visualization of the signals was achieved using Alexa Fluor 488- and 594-conjugated secondary antibodies (Thermo Fisher Scientific), with 4′,6-diamidino-2-phenylindole used to counterstain cell nuclei. The stained sections were examined under a confocal microscope (LSM 900 and 980, Carl Zeiss, Jena, Germany). The liver tissues of human and mice were fixed using 10% (v/v) formalin solution, embedded in paraffin blocks, and sectioned into 5-μm slices for H&E staining. Histological and immunohistochemical analysis of liver tissue areas was quantified using ImageJ software (National Institutes of Health, Bethesda, MD, USA). Some illustrations in this work were created using BioRender (www.biorender.com).

### RNA sequencing

RNA sequencing was conducted to compare mRNA expression levels between orthotopically induced PDX models generated with Matrigel and Liver ECM. mRNA samples were extracted from both Matrigel and Liver ECM xenografts (*n* = 3 per group) using the Qiagen RNeasy Mini Kit. mRNA-Seq was performed by Ebiogen (Ebiogen, Inc., Seoul, South Korea). Library preparation was conducted using the QuantSeq 3′ mRNA-Seq Library Prep Kit (Lexogen GmbH, Vienna, Austria) following the manufacturer’s instructions. The quality of extracted RNA was assessed using a Bioanalyzer 2100 system (Agilent), and a cDNA library was constructed using the NEBNext Ultra II Directional RNA-Seq Kit (NEB). The sequencing was carried out on a NovaSeq 6000 platform, and the sequencing libraries were prepared using the Illumina TruSeq Stranded mRNA Kit (Illumina, San Diego, CA, USA). Data mining and visualization were performed using ExDEGA (Ebiogen Inc.). Gene classification was based on searches conducted using the Database for Annotation, Visualization and Integrated Discovery (DAVID) (http://david.ncifcrf.gov/). Differentially expressed genes (DEGs) were identified based on the criteria of fold change > 2 and *P* value < 0.05. Functional annotation and gene ontology (GO) enrichment analysis were conducted using REVIGO (version 1.8.1). KEGG pathway map was generated using the KEGG Mapper (https://www.genome.jp/kegg/mapper/) (version 5.2). Heatmap visualization of DEGs was generated using GraphPad Prism 10.0 (GraphPad Software, La Jolla, CA, USA).

### Statistical analysis

All statistical analyses were performed using unpaired, 2-sided Student’s *t* tests, and one-way analysis of variance (ANOVA) with Tukey’s honest significant difference post-hoc analysis for multiple comparisons (SPSS 21.0 K for Windows; SPSS, Chicago, IL, USA). Statistical significance was set at *P* < 0.05. GraphPad Prism 10.0 was used for statistical analyses.

## Results

### Establishment of human HCC cell-derived xenograft model using liver ECM

**Fig. 1.**
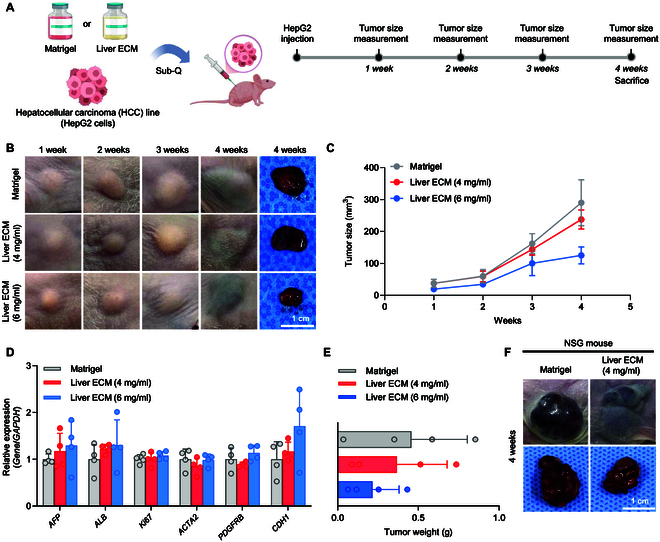
Establishment of xenograft models with the human HCC cell line (HepG2) and liver extracellular matrix (Liver ECM). (A) Schematic illustration of generating subcutaneous xenograft model from the HepG2 cell line. (B) Representative images of tumors over a 4-week period following subcutaneous injection of HepG2 cells encapsulated in either Matrigel or Liver ECM (Regenix; concentrations: 4 and 6 mg/ml) (scale bar = 1 cm). (C) Quantification of tumor size measured every week up to 4 weeks after injection of HepG2 cells encapsulated with Matrigel and Liver ECM (4 mg/ml and 6 mg/ml) (*n* = 4 per group). (D) qPCR analysis of xenograft tumors to compare gene expression levels for liver cancer differentiation markers (*AFP* and *ALB*), proliferation marker (*Ki67*), metastatic markers (*ACTA2* and *PDGFRB*), and tumor tight junction marker (*CDH1*) between Matrigel and Liver ECM (4 and 6 mg/ml) groups at 4 weeks (*n* = 4 per group). (E) Tumor weights at 4 weeks post-injection of HepG2 cells encapsulated with Matrigel or Liver ECM (4 and 6 mg/ml) (*n* = 4 per group). (F) Xenograft models generated by subcutaneous transplantation of HepG2 cells with Matrigel or Liver ECM (Regenix; 4 mg/ml) in immunodeficient NSG mice (scale bar = 1 cm).

To evaluate the feasibility of Liver ECM in developing xenograft models, we first used the human HCC cell line (HepG2 cell). HepG2 cells (1 × 10^6^ cells in 100 μl of hydrogel) encapsulated in Liver ECM hydrogel and Matrigel were subcutaneously injected into the right flank of immunodeficient mice (Balb/c nude) (Fig. 1A and B). To compare the tumorigenic potential of different liver ECM concentrations, we examined the performance of Liver ECM at 2 concentrations (4 and 6 mg/ml) over a 4-week monitoring period (Fig. 1B). The average xenograft tumor volumes measured 4 weeks after transplantation were 290.31, 237.66, and 125.24 mm^3^ for the Matrigel, 4 mg/ml Liver ECM, and 6 mg/ml Liver ECM groups, respectively. The tumor size of the 4 mg/ml Liver ECM group was not significantly different (*P* = 0.3072) from that of the Matrigel group, whereas the 6 mg/ml Liver ECM group showed a substantially smaller tumor size than those 2 groups (Fig. 1B and C). We observed similar expression levels of representative liver cancer differentiation markers (*AFP* and *ALB*), proliferation marker (*Ki67*), tight junction marker (*CDH1*), epithelial-to-mesenchymal transition (EMT), and metastasis-related markers (*ACTA2* and *PDGFRB*) between xenografts established with Matrigel and Liver ECM (Fig. 1D). Four weeks post-injection, liver cancer weights in the 4 and 6 mg/ml liver ECM groups were 95% and 67% relative to the Matrigel group, respectively (Fig. 1E). These findings suggest that xenografts established with 4 mg/ml Liver ECM were comparable to those formed with Matrigel, whereas a higher concentration of Liver ECM (6 mg/ml) was less effective for tumor development. While matrix stiffening is a well-known risk factor that promotes the progression of HCC, the successful initiation of xenograft tumors using patient-derived HCC cells requires a microenvironment that supports robust cancer cell proliferation and tumorigenic capacity. A previous study reported that primary HCC cells proliferate more rapidly and form highly malignant tumors in soft hydrogels, whereas stiff hydrogels lead to smaller tumors with reduced malignancy [18]. This is likely due to the dense polymeric networks in stiff hydrogels, which restrict cell motility and limit material diffusion, thereby suppressing the proliferation of cancer stem cells [19,20]. We compared the mechanical properties of Liver ECM hydrogels at concentrations of 4 and 6 mg/ml and found that the 4 mg/ml hydrogel exhibited a significantly lower elastic modulus than the 6 mg/ml hydrogel (Fig. S1). These findings suggest that a lower-concentration Liver ECM hydrogel (4 mg/ml) may provide a more favorable 3D microenvironment for liver cancer initiation and progression.

Xenograft models have traditionally been established using immunodeficient mice with varying levels of immunosuppression [2]. For instance, Balb/c nude mice lack a thymus, preventing T cell production and are considered immunodeficient, while nonobese diabetic severe combined immunodeficiency (NOD/SCID) mice exhibit more severe immunosuppression due to impaired T and B lymphocyte development, defective natural killer cell function, absent macrophage activity, and reduced complement activity [21,22]. To validate our findings in a more profoundly immunodeficient model beyond Balb/c nude mice, we tested the 4 mg/ml Liver ECM- and Matrigel-based xenograft models in NOD-SCID gamma (NSG) mice (Fig. 1F). Xenografts established in NSG mice developed larger tumors compared to those in immunodeficient Balb/c nude mice over the same 4-week period (Fig. 1F). This result confirms that xenograft models using Liver ECM generate larger tumors in highly immunosuppressive NSG mice, likely owing to higher engraftment rates. Overall, liver cancer xenografts can be effectively established with Liver ECM, comparable to the Matrigel.

### PDX model generated with liver ECM recapitulates patient’s original liver cancer

**Fig. 2.**
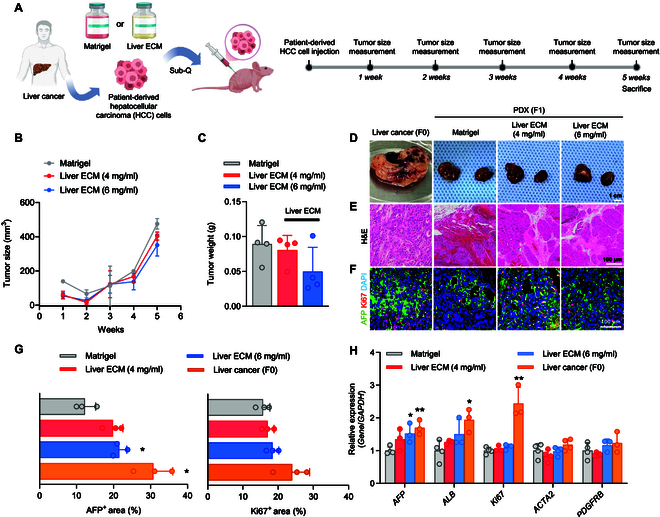
Generation of liver cancer patient-derived xenograft (PDX) models with primary HCC cells and Liver ECM. (A) Schematic illustration of generating the PDX model from patient-derived HCC cells. (B) Tumor growth trajectories measured every week after subcutaneous injection of patient-derived HCC cells mixed with Matrigel or Liver ECM (Regenix; 4 and 6 mg/ml) (*n* = 4 per group). (C) Quantification of tumor weights at 5 weeks after injection of patient-derived HCC cells mixed with Matrigel or Liver ECM (Regenix; 4 and 6 mg/ml) (*n* = 4 per group). (D) Representative images of human liver cancer tissues (F0) and subcutaneous PDX tumors (F1) harvested at 5 weeks post-injection of patient-derived HCC cells mixed with Matrigel or Liver ECM (Regenix; 4 and 6 mg/ml) (scale bar = 1 cm). (E) Hematoxylin and eosin (H&E) staining and (F) immunofluorescence staining showing liver cancer cell marker (AFP) and proliferation marker (Ki67) (scale bar = 100 μm). (G) Quantification of AFP^+^ and Ki67^+^ area (*n* = 3 per group, *P < 0.05 versus Matrigel group) at 5 weeks. (H) qPCR analysis comparing gene expression levels for liver cancer differentiation markers (*AFP* and *ALB*), proliferation marker (*Ki67*), metastasis and EMT-related markers (*ACTA2* and *PDGFRB*) in liver cancer tissue and PDX tumors (Matrigel and Liver ECM groups) at 5 weeks after transplantation (*n* = 3 to 4 per group, *P < 0.05, **P < 0.01 versus Matrigel group).

To further assess the clinical feasibility of PDX models using Liver ECM, patient-derived HCC cells were subcutaneously injected into immunodeficient nude mice (Fig. 2A). We compared PDX models generated with different concentrations of Liver ECM (4 and 6 mg/ml) to those with Matrigel. Five weeks post-injection, the average tumor volumes were 475.08, 403.5, and 350.57 mm^3^ for the Matrigel, 4 mg/ml Liver ECM, and 6 mg/ml Liver ECM groups, respectively (Fig. 2B). This indicates that the 4 and 6 mg/ml Liver ECM groups achieved 85% and 74% of the tumor volume observed in the Matrigel group, respectively. Similarly, tumor weights in the 4 and 6 mg/ml Liver ECM groups reached 88% and 63% of the Matrigel group, respectively (Fig. 2C).

We further compared tumors of the PDX models generated with Liver ECM to the patient’s original tumors to verify the similarity between the HCC xenograft models (5 weeks post-injection) and the patient’s primary liver cancer (Fig. 2D). H&E staining of the patient’s primary liver cancer (F0) revealed high cellular density, pseudoglandular formation with high degree of atypia, and disrupted sinusoidal architecture (Fig. 2E). Similarly, both PDX models generated using Matrigel and Liver ECM exhibited tumor-like features, such as a high degree of atypia, including multinucleated cells, disrupted sinusoidal architecture, and fibrotic stroma, mirroring typical pathology observed in the patient’s liver cancer (Fig. 2E). Immunofluorescence staining showed higher protein expression of the liver cancer differentiation marker AFP in the xenografts generated with Liver ECM (4 and 6 mg/ml) than in those with Matrigel, while the level of the proliferation marker Ki67 was comparable between these groups (Fig. 2F and G). qPCR analysis to check gene expression confirmed similar trends observed in immunofluorescence staining (Fig. 2H). The gene expression of proliferation marker *Ki67* was higher in the primary liver cancer (F0) than in the cancer of PDX models established with Matrigel and Liver ECM though there was no statistical significance among the PDX groups. EMT- and metastasis-related markers (*ACTA2* and *PDGFRB*) were expressed at similar levels in the Matrigel group, the Liver ECM group, and liver cancer tissue. Notably, PDX models generated with Liver ECM displayed higher expression of liver cancer-specific markers *AFP* and *ALB*, compared to PDX models with Matrigel, indicating enhanced tumor progression by Liver ECM. Collectively, PDX models established using Liver ECM displayed histological features, gene expression patterns, and protein profiles resembling those of the patient’s original tumor.

### Liver ECM promotes liver cancer progression and metastasis in an orthotopic PDX model

**Fig. 3.**
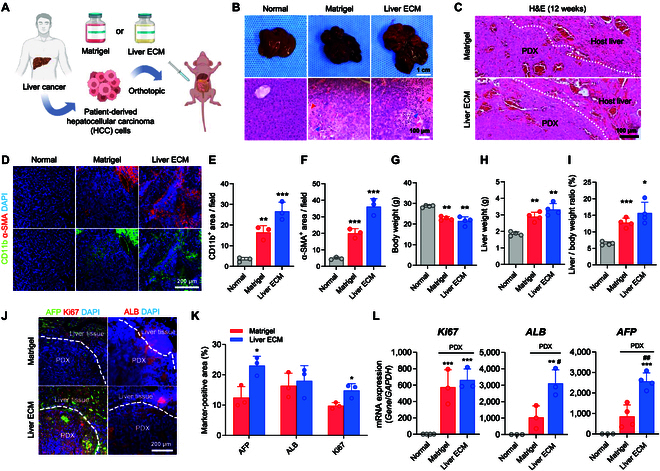
Generation of orthotopic liver cancer PDX models with patient-derived HCC cells and Liver ECM. (A) Schematic illustration of generating orthotopic PDX mouse model with patient-derived HCC cells using Matrigel and Liver ECM. Patient-derived HCC cells were isolated from HCC patient’s liver tissue and injected into the median lobe of the liver. (B and C) Representative liver tissue images (top panels in B) (scale bar = 1 cm) and hematoxylin and eosin (H&E) staining images (bottom panels in B and C) of orthotopically induced xenograft models at 12 weeks post-injection. PDX tumors (Matrigel xenograft and Liver ECM xenograft groups) were compared with normal liver tissue. Blue arrowheads represent intratumoral neutrophils and red arrowheads represent intratumoral fibrosis (scale bars = 100 μm). (D) Immunofluorescence staining images (scale bar = 200 μm) and (E and F) quantification of neutrophil marker (CD11b) and fibrosis marker (α-SMA)-positive area in orthotopic PDX models at 12 weeks post-injection of patient-derived HCC cells (*n* = 3 per group, **P < 0.01, ***P < 0.001 versus Normal liver tissue group). (G to I) Quantification of (G) body weight, (H) liver weight, and (I) liver-to-body weight ratio of orthotopically induced liver cancer xenograft model at 12 weeks post-injection (normal liver tissue, Matrigel xenograft, and Liver ECM xenograft groups; *n* = 4 per group, *P < 0.05, **P < 0.01, ***P < 0.001 versus Normal liver tissue group). (J) Immunofluorescence staining images (scale bar = 200 μm) and (K) quantification of liver cancer differentiation markers (AFP and ALB) and proliferation marker (Ki67)-positive area in orthotopic PDX models at 12 weeks post-injection of patient-derived HCC cells (*n* = 3 per group, *P < 0.05 versus Matrigel xenograft group). (L) qPCR analysis comparing gene expression levels in normal liver tissue, Matrigel xenograft, and Liver ECM xenograft groups for liver cancer differentiation markers (*AFP* and *ALB*) and proliferation marker (*Ki67*) at 12 weeks (*n* = 3 to 4 per group, **P < 0.01, ***P < 0.001 versus Normal liver tissue, #*P* < 0.05, ##*P* < 0.01 versus Matrigel xenograft).

To evaluate the feasibility of Liver ECM for generating the orthotopic PDX model—which is highly metastatic and more closely resembles patient’s primary tumors than the subcutaneous PDX model [23,24]—we directly injected HCC cells derived from patient tumors into the liver tissue of immunodeficient nude mice (Fig. 3A). A Liver ECM concentration of 4 mg/ml, optimized from previous experiments, was used to establish orthotopic HCC xenografts, which were compared to those generated via orthotopic injection with Matrigel and normal liver tissue (Fig. 3A and B). H&E staining showed that the PDX tumors formed with Matrigel and Liver ECM displayed proliferative tumor cells, intratumoral fibrosis, and the presence of neutrophils, which were not observed in normal liver tissue (Fig. 3B and C). Additionally, we observed a higher degree of CD11b^+^ neutrophil infiltration and α-SMA^+^ intratumoral fibrosis in the Liver ECM group compared to the Matrigel group (Fig. 3D to F). These findings confirm the successful establishment of well-differentiated xenograft models that effectively recapitulate the disease phenotypes observed in liver cancer patients. Measurement of body weights 12 weeks post-injection revealed that the mice of the Matrigel group experienced weight loss as liver cancer developed and those of Liver ECM group underwent even greater weight loss (Fig. 3G). Liver weights in both the Matrigel and Liver ECM groups were higher than in the normal group, with the Liver ECM group exhibiting the highest liver weight, which was attributed to the highly proliferative tumor mass (Fig. 3H). Accordingly, we could observe the greatest increase in the liver-to-body weight ratio of Liver ECM xenografts (Fig. 3I). Immunostaining for liver cancer differentiation markers (AFP and ALB) and the proliferation marker (Ki67) demonstrated that the orthotopic PDX tumors of the Liver ECM group showed greater progression compared to those of the Matrigel group (Fig. 3J and K). These findings were confirmed with the results from qPCR analysis to check gene expression levels (Fig. 3L), where *AFP* and *ALB* expression were significantly much higher in the Liver ECM group than in the Matrigel group, along with a modest increase in *Ki67* expression. Overall, our data demonstrate that Liver ECM promotes tumorigenesis in an orthotopic xenograft model.

**Fig. 4.**
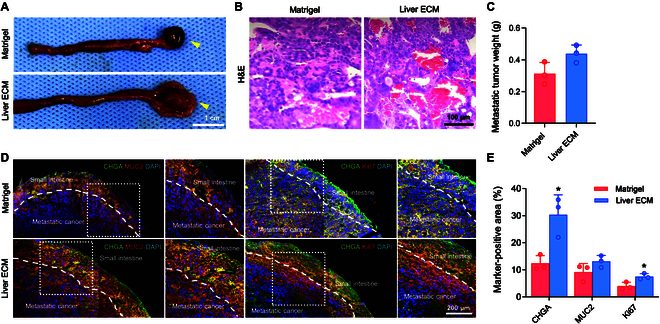
Liver ECM enhances metastatic potential of liver cancer xenograft to small intestine. (A) Photographs and (B) hematoxylin and eosin (H&E) staining images showing metastasis tumors in small intestine of orthotopically induced PDX liver cancer model 12 weeks post-injection of patient-derived HCC cells using Matrigel and Liver ECM (scale bar = 100 μm). (C) Quantification of metastatic tumor weight in orthotopic PDX model at 12 weeks post-injection (Matrigel and Liver ECM xenograft groups; *n* = 3 per group). (D) Immunofluorescence staining for intestine cancer markers (CHGA and MUC2) and proliferation marker (Ki67) (scale bar = 200 μm) and (E) quantification of intestine cancer marker (CHGA and MUC2)- and proliferation marker (Ki67)-positive area in small intestine tissue of the orthotopic PDX model at 12 weeks post-injection (Matrigel and Liver ECM xenograft groups; *n* = 3 per group, *P < 0.05 versus Matrigel xenograft group).

Next, we investigated metastasis in orthotopic PDX liver cancer models established with Matrigel and Liver ECM. Metastasis to small intestine was observed in both 12-week PDX models generated using Matrigel and Liver ECM (Fig. 4A). Intestinal metastasis of HCC is relatively uncommon in clinical settings. However, several preclinical studies have reported that it can occur through blood-borne or direct invasion to adjacent organs, including the intestine, particularly in models characterized by high invasiveness and metastatic potential [25,26]. HCC can spread to the intestines via the bloodstream, especially in the presence of portal vein tumor thrombus, or through the lymphatic system [27]. Based on this precedent and our own preliminary observations, we included the intestine as one of the primary sites for metastasis analysis. Metastatic tumors in PDX models with Liver ECM exhibited increased volume compared to those with Matrigel. H&E staining revealed varying degrees of pleomorphism, high-grade intramucosal neoplasia, and necrosis in both Matrigel and Liver ECM groups (Fig. 4B). The average weight of metastatic tumors in the intestine was higher in the Liver ECM group than in the Matrigel group (Fig. 4C). Immunostaining showed that intestinal cancer markers (MUC2 and CHGA) and proliferation marker (Ki67) were highly expressed in metastatic tumors derived from orthotopic PDX liver cancers established with Liver ECM (Fig. 4D and E). To confirm the hepatic origin of these metastatic lesions, we conducted immunohistochemical staining for liver cancer-related markers (ALB and AFP) with human-specific antigens (human GAPDH and human nuclear antigen) in the metastatic lesions (Fig. S2). These markers were strongly expressed in the intestinal nodules, confirming their derivation from the transplanted human HCC cells. These results confirm that the intestinal metastatic lesions share the same hepatic phenotype as the human primary HCC, thereby supporting the interpretation of enhanced metastatic behavior in our orthotopic xenograft model. Overall, while the subcutaneous xenograft model did not show substantial differences between the Matrigel and Liver ECM groups, the orthotopically induced liver cancer xenograft model using Liver ECM produced highly differentiated tumors with greater volume and increased intestinal metastasis compared to the model using Matrigel. These results suggest that Liver ECM capable of providing liver-specific microenvironment can facilitate integration of transplanted HCC cells more effectively into liver tissue, thereby promoting proliferation, differentiation, and metastasis of liver cancer cells in the orthotopic PDX models.

### Transcriptomic analysis reveals that liver ECM recapitulates liver cancer characteristics in orthotopically induced PDX model

**Fig. 5.**
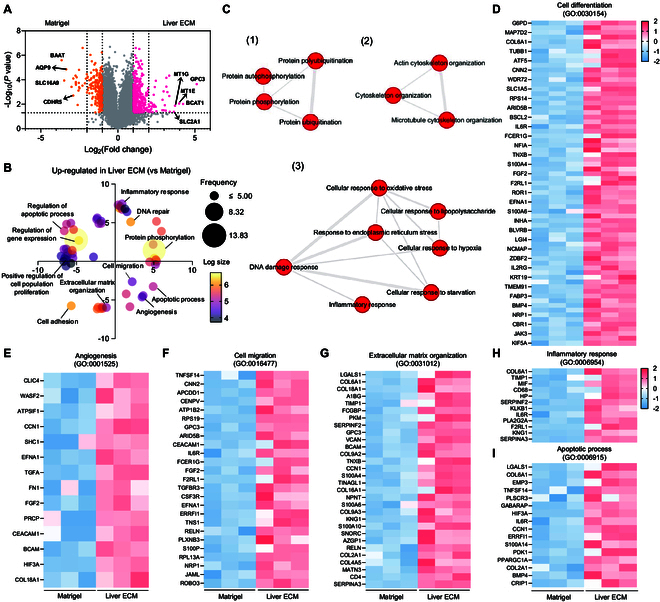
RNA sequencing analysis demonstrates that Liver ECM closely recapitulates liver cancer characteristics in orthotopic PDX models. (A) Volcano plot showing significantly differentially expressed genes (DEGs) of Matrigel xenograft groups versus Liver ECM xenograft groups (*n* = 3 per group, enrichment fold change ≥ 2, *P* < 0.05). (B) The GOBP analysis for DEGs up-regulated in Liver ECM xenograft group. Color represents the *P* value, and the circle size indicates the frequency. (C) GO term analysis using interactive graphs showing up-regulated GO terms in Liver ECM xenograft group compared to the Matrigel xenograft group. The circle size corresponds to the *P* value for the GO term. (D to I) Heatmaps displaying DEG profiles related to (D) cell differentiation (GO:0030154), (E) angiogenesis (GO:0001525), (F) cell migration (GO:0016477), (G) extracellular matrix organization (GO:0031012), (H) inflammatory response (GO:0006954), and (I) apoptotic process (GO:0006915) in the Matrigel xenograft group and the Liver ECM xenograft group using row *z*-score normalization. Color bar represents the *z*-score values of gene expression level.

To investigate how Liver ECM promotes liver cancer progression and enhances its resemblance to actual liver cancer tissue, we conducted a transcriptomic analysis comparing the PDX orthotopic model generated with Liver ECM to that induced with Matrigel (Fig. 5 and Figs. S3 and S4). A volcano plot identified glypican-3 (*GPC3*) as one of the most significant DEGs in the Liver ECM group (Fig. 5A), a key molecular marker associated with aggressive liver cancer and widely used for its diagnosis [28]. Additionally, branched-chain amino acid transaminase 1 (*BCAT1*) and solute carrier family 2 member 1 (*SLC2A1*)—genes known to promote cancer cell invasion [29] and tumor immune infiltration [30], respectively—were also up-regulated in the Liver ECM group. In contrast, the Matrigel group showed higher expression of genes with indirect relevance to liver cancer, such as bile acid-CoA amino acid N-acyltransferase (*BAAT*), which plays a role in bile acid production [31]. Furthermore, aquaporin 9 (*AQP9*), known to inhibit liver cancer growth and metastasis [32], and cadherin-related family member 5 (*CDHR5*), which suppresses HCC cell proliferation [33], were up-regulated in the Matrigel group (Fig. 5A).

To gain deeper insights into the biological roles of the DEGs in the Liver ECM group, we performed gene ontology biological process (GOBP) analysis, revealing significant enrichment in pathways related to cell migration, angiogenesis, apoptosis, and inflammatory response—major processes closely linked to liver cancer progression (Fig. 5B). GO term analysis using interactive graphs identified major categories of up-regulated interactions in the Liver ECM group: (1) protein phosphorylation and ubiquitination, (2) ECM-related cytoskeletal organization, and (3) DNA damage response associated with oxidative stress, inflammation, and hypoxia (Fig. 5C). Further GO term analysis of genes significantly up-regulated in the Liver ECM group highlighted strong interactions with key liver cancer-related pathways, including the mitogen-activated protein kinase (MAPK) cascade [34], extracellular signal-regulated kinase 1/2 (ERK 1/2) cascade [35], and Wnt signaling [36] (Fig. S3). Additionally, biological processes related to cell migration, angiogenesis, and apoptosis were also significantly enriched in the Liver ECM xenografts compared to those in Matrigel xenografts (Fig. S3). Heatmap analysis identified key DEGs associated with cell differentiation (GO:0030154), angiogenesis (GO:0001525), cell migration (GO:0016477), extracellular matrix organization (GO:0031012), inflammatory response (GO:0006954), and apoptotic process (GO:0006915), all of which were significantly up-regulated in Liver ECM xenografts (Fig. 5D to I). Notably, Kyoto Encyclopedia of Genes and Genomes (KEGG) pathway analysis revealed that hypoxia-inducible factor 1 (HIF1) signaling was markedly up-regulated in the Liver ECM group, with increases in pathways associated with inflammation, angiogenesis, anaerobic metabolism, and the regulation of proliferation and apoptosis (Fig. S4). Collectively, these findings demonstrate that PDX models generated with Liver ECM displayed a marked increase in biological functions related to angiogenesis, hypoxia, inflammatory response, and cell migration, reinforcing its ability to more effectively recapitulate liver cancer characteristics.

While HCC often exhibits mesenchymal and invasive features especially in advanced stages, its key epithelial characteristics are important for accurately modeling liver cancer. To assess whether our orthotopic PDX models preserve these epithelial features, we performed immunohistochemical staining for representative epithelial markers including E-cadherin (ECAD), cytokeratin 19 (CK19/KRT19), and cytokeratin 18 (CK18/KRT18) on liver cancer tissue sections from orthotopic xenografts (Fig. S5A). The staining results demonstrate robust expression of these epithelial markers in the Liver ECM group, indicating preservation of epithelial identity despite the acquisition of mesenchymal features. We also confirmed that DEGs associated with epithelial cell proliferation (GO:0050678) were highly expressed in Matrigel- and Liver ECM-derived xenografts, indicating preservation of epithelial characteristics in both models. For example, *CEACAM1* and *MARVELD3*, involved in epithelial polarity and tight junction maintenance, respectively [37,38], were up-regulated in the Liver ECM group, whereas *LRG1* and *KLF9*, involved in epithelial cell proliferation and maintenance [39,40], showed higher expression in the Matrigel group (Fig. S5B). This transcriptomic evidence aligns with the immunohistochemical staining data and confirms that epithelial features are well retained in the Liver ECM-based xenograft models.

### Liver ECM enhances progression of liver cancer organoids and development of subcutaneous PDOX

**Fig. 6.**
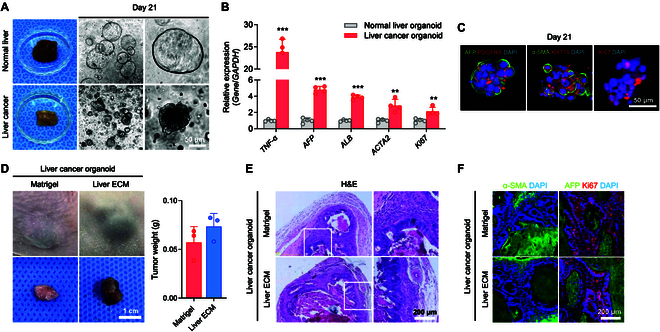
Liver ECM facilitates establishment of the highly differentiated patient-derived organoid xenograft (PDOX) model. (A) Bright-field images showing normal liver organoids and liver cancer organoids generated in Matrigel at day 21 of culture (scale bar = 50 μm). (B) qPCR analysis to compare gene expression levels for inflammation (*TNF-a*), liver cancer differentiation (*AFP* and *ALB*), metastasis and EMT-related marker (*ACTA2*), and proliferation (*Ki67*) markers in organoids from human normal liver and HCC patient liver (*n* = 3 to 4 per group, **P < 0.01, ***P < 0.001 versus normal liver organoids). (C) Immunofluorescence staining images of liver cancer organoids for liver cancer differentiation markers (AFP and KRT19), metastasis and epithelial-to-mesenchymal transition (EMT)-related markers (PDGFRB, a-SMA), and proliferation marker (Ki67) (scale bar = 50 μm). (D) Photographs of the subcutaneous xenograft tumors (scale bar = 1 cm) and quantification of tumor weight at 12 weeks post-injection of patient-derived liver cancer organoids with Matrigel or Liver ECM (Regenix; 4 mg/ml). (E) Hematoxylin and eosin (H&E) staining images of Matrigel PDOX and Liver ECM PDOX models (scale bar = 200 μm). (F) Immunofluorescence staining images of the sectioned PDOX tumors for liver cancer differentiation marker (AFP), EMT-related marker (α-SMA), and proliferation marker (Ki67) at 12 weeks (scale bar = 200 μm).

Finally, we tested the Liver ECM for establishment of the xenograft models with patient-derived liver cancer organoids. To assess the potential of the Liver ECM in developing a PDOX model, we compared human normal liver organoids and patient-derived liver cancer organoids in terms of morphology and liver cancer-associated gene expression patterns (Fig. 6). Normal liver organoids and patient-derived liver cancer organoids were produced via 3D culture with liver ductal cells and patient-derived HCC cells, respectively (Fig. 6A). qPCR analysis revealed that the gene expression of liver cancer-specific differentiation markers *AFP* and *ALB* was much higher in patient-derived liver cancer organoids (5-fold and 4-fold, respectively) than in normal liver organoids (Fig. 6B). The expression of inflammation-related marker *TNF-α*, EMT-related marker *ACTA2*, and proliferation marker *Ki67* was approximately 25-fold, 3-fold, and 2-fold higher in patient-derived liver cancer organoids, respectively, compared to normal liver organoids (Fig. 6B). We then confirmed that liver cancer organoids expressed liver cancer-specific characteristics by checking the expression of liver cancer differentiation markers (AFP and KRT19), proliferation marker (Ki67), and EMT activation markers (PDGFRB and α-SMA) (Fig. 6C). These findings imply that liver cancer organoids can replicate the proliferation, differentiation, and metastasis of human liver cancer.

To facilitate the establishment of the PDOX model, we applied Liver ECM for subcutaneous transplantation of patient-derived liver cancer organoids (Fig. 6D to F). Compared with the PDOX model established with Matrigel, PDOX tumors induced by Liver ECM showed a much larger size 12 weeks after transplantation (Fig. 6D). In H&E staining, both PDOX groups exhibited HCC phenotypes with intratumoral fibrosis and high degree of nuclear atypia (Fig. 6E). Immunostaining analysis revealed notably increased expression levels of liver cancer marker (AFP), proliferation marker (Ki67), and EMT activation marker (α-SMA) in both PDOX models generated with Matrigel and Liver ECM (Fig. 6F), confirming the successful establishment of PDOX models of liver cancer organoids using Liver ECM to the levels comparable to Matrigel.

## Discussion

In this study, we developed a liver cancer PDX model using decellularized liver ECM, which provides a liver tissue-specific microenvironment, as an alternative to the conventional matrix such as Matrigel. Traditional PDX models generated with Matrigel require further advancements in growth, differentiation, and metastasis of liver cancer as Matrigel does not replicate the complex liver tissue-specific microenvironment. Consequently, there is an urgent need for a matrix capable of producing liver cancer xenograft models that closely mimic actual patient tumor tissue. The decellularized liver tissue-derived matrix is known to contain various liver-specific ECM components and secreted factors [14,15]. Previous studies have consistently demonstrated that hepatocytes, liver cancer cells, and stem cell-derived liver organoids exhibit enhanced growth and differentiation in ECM hydrogels derived from decellularized liver tissue [14,41].

Here, we identified the optimal concentrations of Liver ECM hydrogels for efficient generation of liver cancer xenograft models (Figs. 1 and 2). Our results demonstrated that PDX models established using Liver ECM achieved development and progression of liver cancer tissue at levels similar to or even better than those established with the conventional matrix, Matrigel (Figs. 2 to 4). Notably, PDX models derived from primary HCC cells and Liver ECM retained the protein and gene expression profiles of the parental patient tumors (Fig. 2F to H), enabling more accurate clinical predictions and advancing the development of personalized cancer therapies. Importantly, orthotopic PDX models established with Liver ECM at optimal conditions could represent primary patient-derived liver cancer better than those with Matrigel (Fig. 3). Incorporating Liver ECM into the orthotopic xenograft model might enhance tumor–stromal interactions between the intrahepatic xenograft and host tissue. This feature of Liver ECM resulted in improved growth, larger volumes, higher differentiation, and increased metastatic potential of tumors in a liver-specific orthotopic xenograft model (Figs. 3 and 4), offering a better representation of human tumor pathophysiology. Our transcriptomic analysis revealed that these effects of Liver ECM are mediated through pathways associated with angiogenesis, hypoxia, inflammation, and cell migration (Fig. 5 and Figs. S3 and S4). In this study, we also successfully established PDOX models using patient-derived liver cancer organoids and Liver ECM (Fig. 6). Our study underscores the biological and clinical relevance of PDOX models constructed with Liver ECM as an alternative to Matrigel.

The PDOX model holds substantial promise for translational oncology by enabling patient-specific in vivo drug efficacy testing. By preserving the molecular and phenotypic characteristics of the original tumor, it enables accurate assessment of chemotherapeutic and targeted agents [5,6]. Integration of molecular profiling with treatment outcomes supports biomarker discovery and advances precision oncology. In liver cancer, incorporating liver-specific ECM further can enhance the model’s fidelity for studying microenvironment-driven tumor behavior and therapeutic responses. Despite their advantages, PDOX models have several limitations. One challenge is the inconsistent efficiency of organoid generation and engraftment, as not all patient tumors form organoids or establish xenografts, potentially biasing the model toward more aggressive clones and limiting its applicability across diverse patient populations [5,42]. The labor-intensive, time-consuming nature of PDOX establishment, along with the need for specialized expertise, restricts scalability for high-throughput applications. While more physiologically relevant than 2D models, PDOX systems still lack full recapitulation of stromal diversity, vascularization, and immune interactions [5,42]. Moreover, extended in vitro culture may induce genetic or epigenetic alterations, reducing model fidelity for drug testing and biomarker discovery.

In this study, we focus on the roles of tissue-specific ECM in improving xenograft models, but need to consider incorporation of additional cellular components of TME. The liver TME consists of the ECM, immune cells, proinflammatory cells, cancer-associated fibroblasts (CAFs), hepatic stellate cells (HSCs), and vascular endothelial cells [43]. Recent research highlights the TME’s critical role in tumorigenesis, emphasizing its bidirectional interactions with tumors, which are pivotal in driving tumor growth, metastasis, and progression [44]. Various TME components, particularly tumor-promoting CAFs, HSCs, and immune cells, markedly influence tumor hallmarks, often through ECM remodeling [45,46]. These alterations in cellular diversity lead to TME-mediated changes in ECM architecture [47]. Understanding the dynamic remodeling of the ECM during liver cancer progression is crucial, and there is an urgent need to develop TME-integrated PDX or PDOX models [47,48]. Given that Liver ECM demonstrates strong protein–protein interactions—such as ECM remodeling, organization, and cell adhesion—that are closely linked to tumorigenesis through critical crosstalk with TME cells [49], it is imperative to create advanced xenograft models that integrate Liver ECM with tumor-associated microenvironmental cells, capable of addressing the limitations of current xenograft models.

## Data Availability

All data generated or analyzed in this study are available within the article and Supplementary Materials. Additional information can be obtained from the corresponding authors upon reasonable request.
