# Supplementary material for: Liver-Specific Extracellular Matrix Enables High-Fidelity Patient-Derived Hepatocellular Carcinoma Xenograft Models
Source: Biomater Res. 2025 Aug 21;29:0242. doi: 10.34133/bmr.0242 (PMC12369845; doi:10.34133/bmr.0242)
Supplement: Supplementary 1 — Figs. S1 to S5 [file bmr.0242.f1.docx]

Supplementary Information

**Liver-specific extracellular matrix enables high-fidelity patient-derived hepatocellular carcinoma xenograft models**

Su Kyeom Kim^1^, Jungho Bae^1^, Mi Jeong Lee^1^, Dai Hoon Han^2*^, and Seung-Woo Cho^1,3,4*^

^1^ Department of Biotechnology, Yonsei University, Seoul 03722, Republic of Korea

^2^ Department of Surgery, Yonsei University College of Medicine, Seoul 03722, Republic of Korea

^3^ Cellartgen, Seoul 03722, Republic of Korea

^4^ Center for Nanomedicine, Institute for Basic science (IBS), Seoul 03722, Republic of Korea

***Corresponding authors**

Prof. Seung-Woo Cho

Department of Biotechnology, Yonsei University, 50 Yonsei-ro, Seodaemun-gu, Seoul 03722, Republic of Korea. E-mail: [seungwoocho@yonsei.ac.kr](mailto:seungwoocho@yonsei.ac.kr)

Prof. Dai Hoon Han

Department of Surgery, Yonsei University College of Medicine, 50-1 Yonsei-ro, Seodaemun-gu, Seoul 03722, Republic of Korea. E-mail: dhhan@yuhs.ac


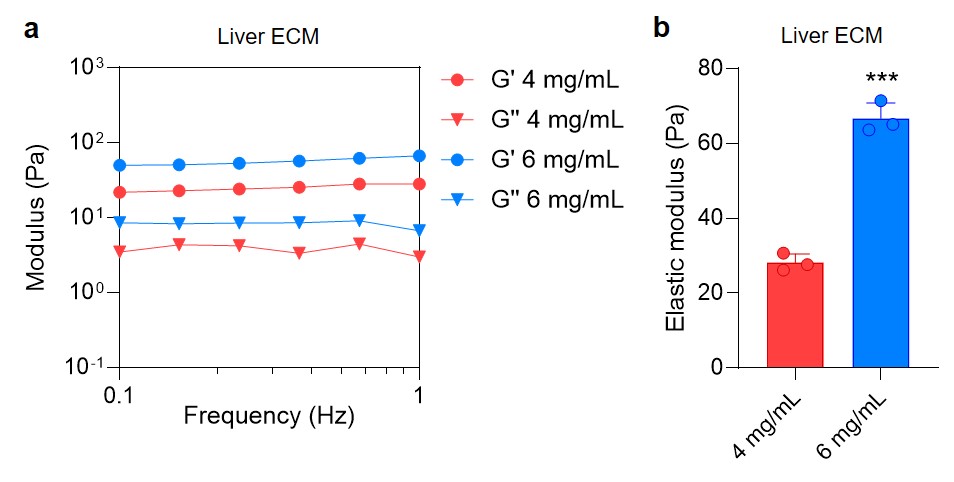


**Supplementary Figure 1. Characterization of the mechanical properties of Liver ECM hydrogels.** Rheological analysis to measure (**a**) the storage (G’) and loss (G”) moduli and (**b**) the average elastic modulus of Liver ECM hydrogels at 4 and 6 mg/mL concentrations (*n* = 3 per group, ****p* < 0.001 versus 4 mg/mL group).


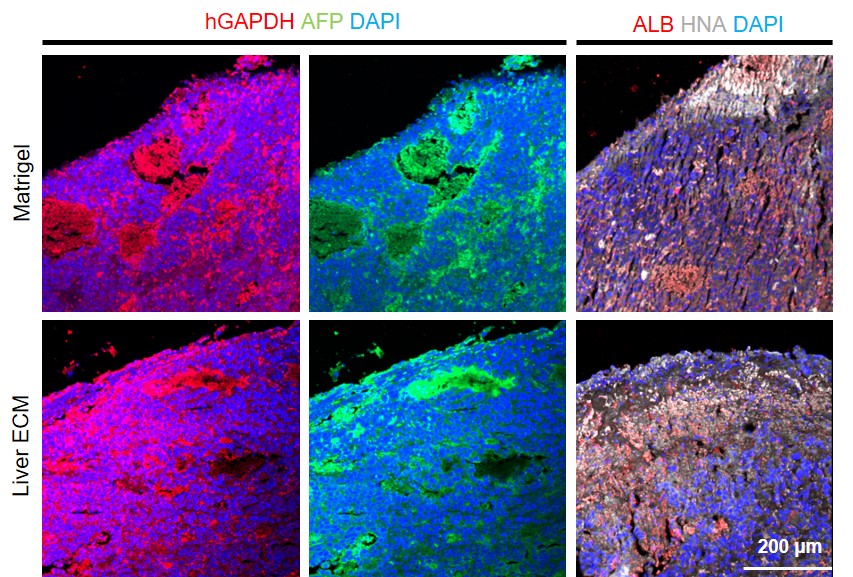


**Supplementary Figure 2. Validation of the hepatic origin of small intestinal metastatic lesions in orthotopic PDX models generated with patient-derived HCC cells.** Immunofluorescence staining of liver cancer differentiation markers (AFP, ALB) and human-specific antigens (human GAPDH; hGAPDH, human nuclear antigen; HNA) in metastatic regions in small intestine tissue of the orthotopic PDX model at 12 weeks post-injection (scale bar = 200 μm).


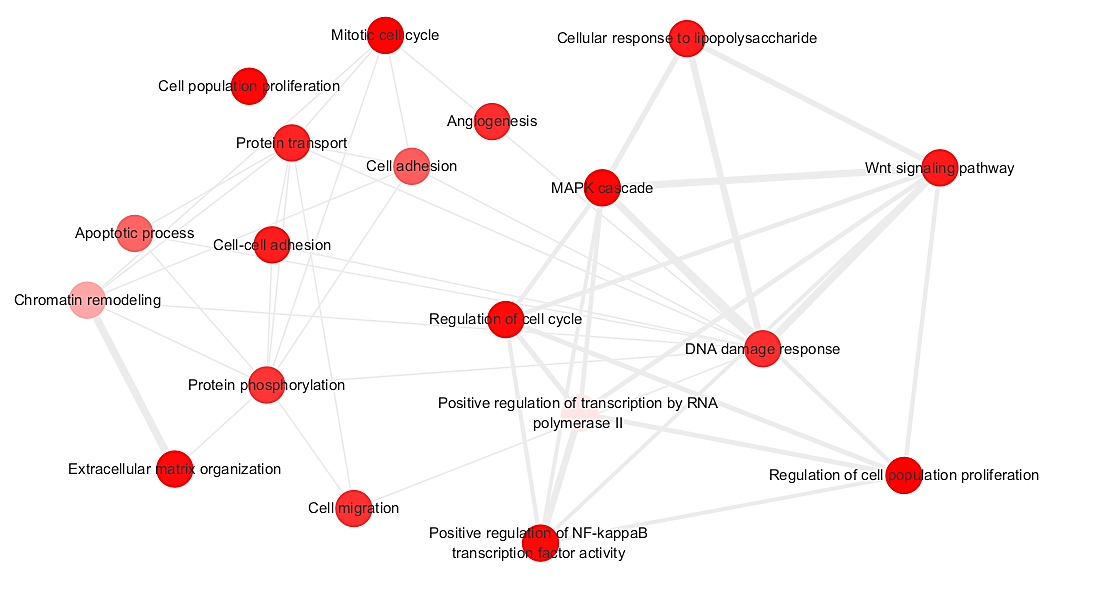


**Supplementary Figure 3. Gene ontology (GO) term analysis upregulated in orthotopic Liver ECM xenograft models.** Interactive graph showing upregulated GO terms in Liver ECM xenograft groups compared to Matrigel xenograft groups (*n* = 3 per group). The circle size corresponds to the *p* value for the GO term.


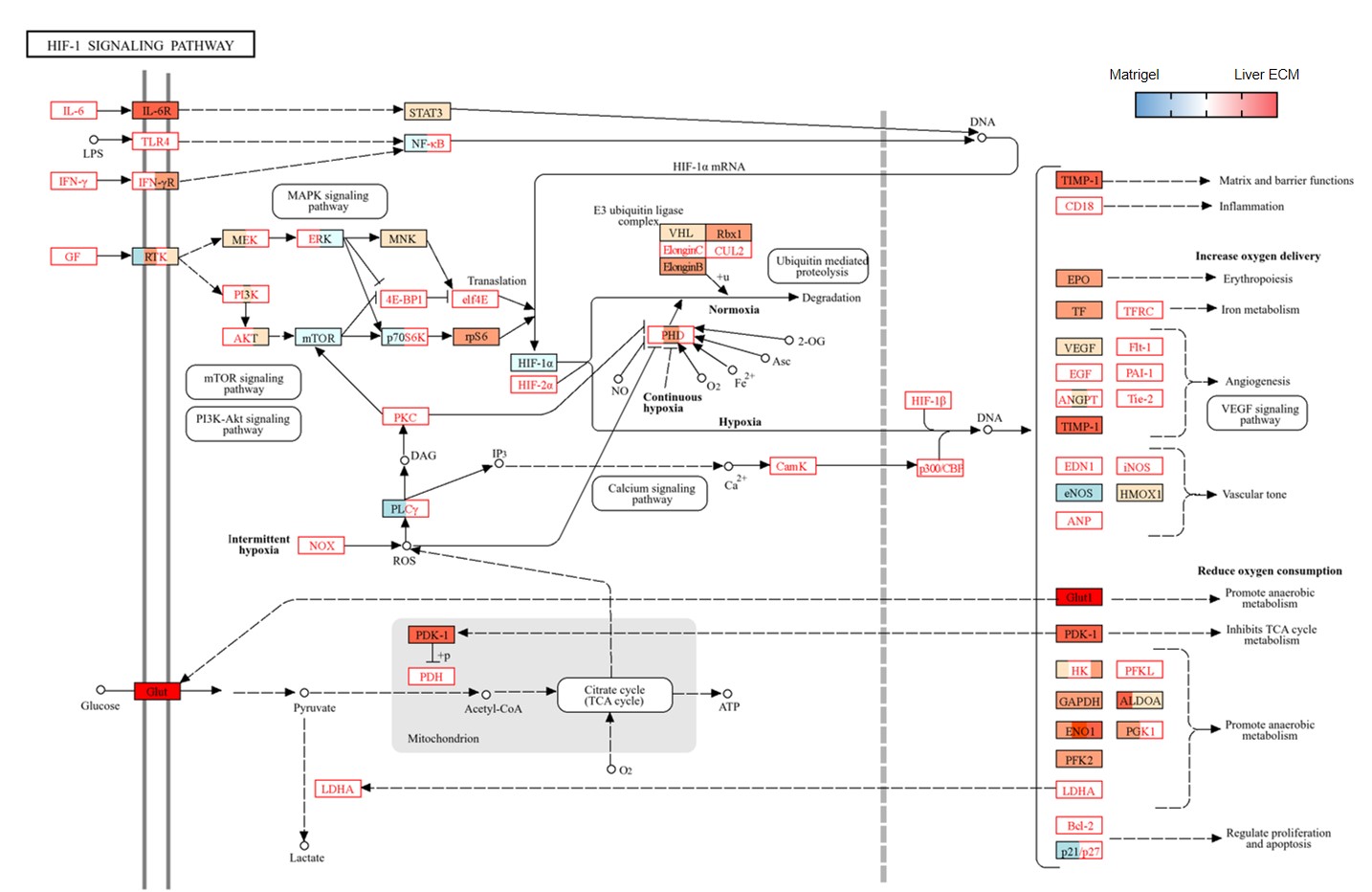


**Supplementary Figure 4. KEGG pathways enrichment analysis comparing orthotopic Matrigel and Liver ECM xenograft models.** Kyoto Encyclopedia of Genes and Genomes (KEGG) pathways enrichment analysis of the HIF-1 signaling pathway to compare orthotopic xenograft models generated with Matrigel and Liver ECM (*n* = 3 per group). Red color indicates pathways more strongly associated with Liver ECM group compared to Matrigel group, while blue color represents pathways more closely related to Matrigel group than Liver ECM groups.


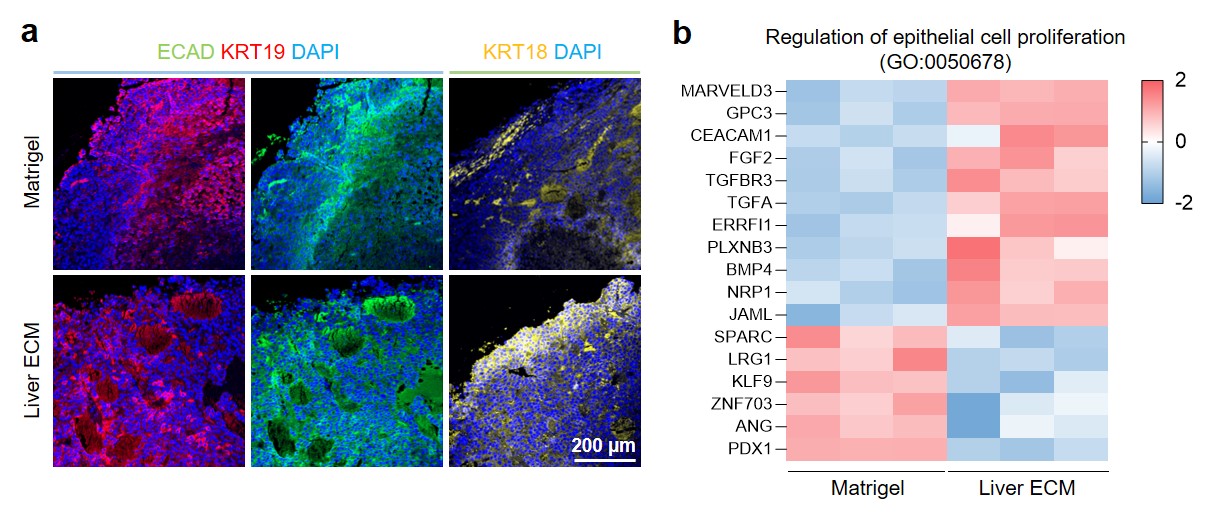


**Supplementary Figure 5. Orthotopic PDX models maintain epithelial identity despite mesenchymal transition.** (**a**) Immunofluorescence staining of epithelial cell markers (ECAD, KRT19, KRT18) in the tumors from orthotopic PDX models at 12 weeks post-injection of patient-derived HCC cells (scale bar = 200 μm). (**b**) Heatmap displaying DEG profiles related to regulation of epithelial cell proliferation (GO:0050678) in Matrigel and Liver ECM xenograft groups using row z-score normalization. Color bar represents the z-score values of gene expression level.
